# SOR: project methodology

**DOI:** 10.1054/bjoc.2000.1757

**Published:** 2001-05

**Authors:** B Fervers, J Hardy, M P Blanc-Vincent, S Theobald, A Bataillard, F Farsi, G Gory, S Debuiche, S Guillo, J L Renaud-Salis, R Pinkerton, P Bey, T Philip

**Affiliations:** Standards Options et Recommandations, Fédération Nationale des Centres de Lutte Contre le Cancer, Paris, France; Centre Léon Bérard, Lyon, France; Royal Marsden NHS Trust, Surrey, UK; Institut Bergonié, Bordeaux, France; Centre Jean Godinot, Reims, France; Centre Val d'Aurelle, Montpellier, France; Centre Alexis Vautrin, Nancy, France


British Journal of Cancer (2001) **84** (Supplement 2), 8–16
© 2001 FNCLCC
doi: 10.1054/ bjoc.2001.1757, available online at http://www.idealibrary.com on IDEAL®

### SOR: project methodology

**B Fervers[1,2], J Hardy[1,3], MP Blanc-Vincent[1,4], S Theobald[5], A Bataillard[1,2], F Farsi[2], G Gory[1,6], S Debuiche[1], S Guillo[1], JL Renaud-Salis[4], R Pinkerton[3], P Bey[7] and T Philip[1,2]**

[1]Standards, Options et Recommandations, Fédération Nationale des Centres de Lutte Contre le Cancer, Paris, France; [2]Centre Léon Bérard, Lyon, France; [3]Royal Marsden NHS Trust, Surrey, UK; [4]Institut Bergonié, Bordeaux, France; [5]Centre Jean Godinot, Reims, France; [6]Centre Val d'Aurelle, Montpellier, France; [7]Centre Alexis Vautrin, Nancy, France

Clinical practice guidelines (CPGs) have been defined as 'systematically developed statements to assist practitioner and patient decisions about appropriate healthcare for specific clinical circumstances' (Institute of Medicine, 1990). The aim is to inform the clinician and the patient of the current best clinical practice in a given situation, based on a synthesis of the available evidence. The 'Standards, Options and Recommendations' (SOR) project develops evidence-based CPGs in oncology and has been undertaken by the French National Federation of Cancer Centers (FNCLCC) since 1993 (Fervers et al, 1995). The project is a collaboration between the FNCLCC (a Federation of the 20 French comprehensive cancer centres) and specialists from French public universities, general hospitals and private clinics. The aim of the project is to increase the quality and efficiency of care given to cancer patients by developing, disseminating and implementing CPGs. SORs are designed to provide a decision guide for cancer specialists in the choice of strategies for the diagnosis, treatment and follow-up of cancer patients.

The FNCLCC represents the regional cancer centres (CRCCs) as a collaborative body at national and international level and coordinates activity in scientific, economic and social domains. The CRCCs are public service hospitals and have a mission of multidisciplinary cancer care, education and research. Twenty-five percent of all cancer patients in France (240 000 new cases every year) are managed within the CRCCs. The remainder are treated in public universities, general hospitals and private clinics.

The SOR project encompasses the progressive development of CPGs for the initial management of cancer in adults and children, for supportive care and control of symptoms in cancer patients and for the standardization of 'good clinical practice' throughout the various disciplines involved in cancer care. It has also undertaken the development of CPGs specifically for nursing and paramedical staff, as well as the provision of evidence-based information for patients.

### GUIDELINE DEVELOPMENT PROCESS

The guideline development process is based on literature review and critical appraisal by specialists from the CRCCs, the French public universities, general hospitals and private clinics, with feedback from practitioners in cancer care delivery. The methodology and development process were defined in 1993 by the FNCLCC (Fervers et al, 1995) based on the experience of various organizations who have been involved in guideline development, in particular the French National Agency for Accreditation and Evaluation in Health (ANAES, formerly ANDEM) (Agence Nationale pour le Développement de l'Evaluation Médicale, 1993; Agency for Health Care Policy and Research, 1992). ANAES, a government-funded agency, develops CPGs for various domains at the national level.

The methodological approach combines systematic review with expert judgement, rather than methods based entirely on either scientific data or the opinion of experts (Mulrow, 1987; Woolf, 1992; Fervers et al, 1995; Browman et al, 1995). It thus provides qualitative systematic reviews as compared to quantitative systematic reviews or meta-analyses.

The guideline development process identifies eight separate steps prior to final approval: topic selection, formation of a working group, defining the questions, literature search, critical appraisal, synthesis of the evidence, formulation of the recommendations and finally independent, external review (Fervers, 2000).

### Topic selection

The SOR project covers as a priority all aspects of first-line management (including diagnosis, staging, prevention, first-line treatment and follow-up) in all cancers, adult and paediatric. This is the basis for topic selection by the executive committee of the SOR project, in collaboration with multidisciplinary disease site groups and the scientific committee of the SOR program. In addition, the CRCCs, ANAES or scientific societies can commission the SOR programm to develop cancer guidelines for specific topics.

### Working groups

The working groups for each subject are multidisciplinary, made up of experts from the specialties involved in the management of a given tumour type or a specific topic. This includes representatives from the CRCCs, the French public universities, general hospitals, private clinics and scientific societies. Each working group is assisted by a methodologist who contributes to and coordinates the drafting and editing SOR process, along with the organization of working group meetings. The methodologists form part of the organizational group of the SOR program (COSOR) and report to a central project coordinator, who in turn sits on the executive committee of the SOR project (see Figure 1).

### Refining the questions

The first task of each group is to define specific and relevant questions for each clinical situation considered. This includes a definition of objectives, the population to be considered, the potential diagnostic methods or therapeutic interventions utilized and the criteria with which to evaluate the interventions considered. This forms the basis of the definition of the literature search strategy.

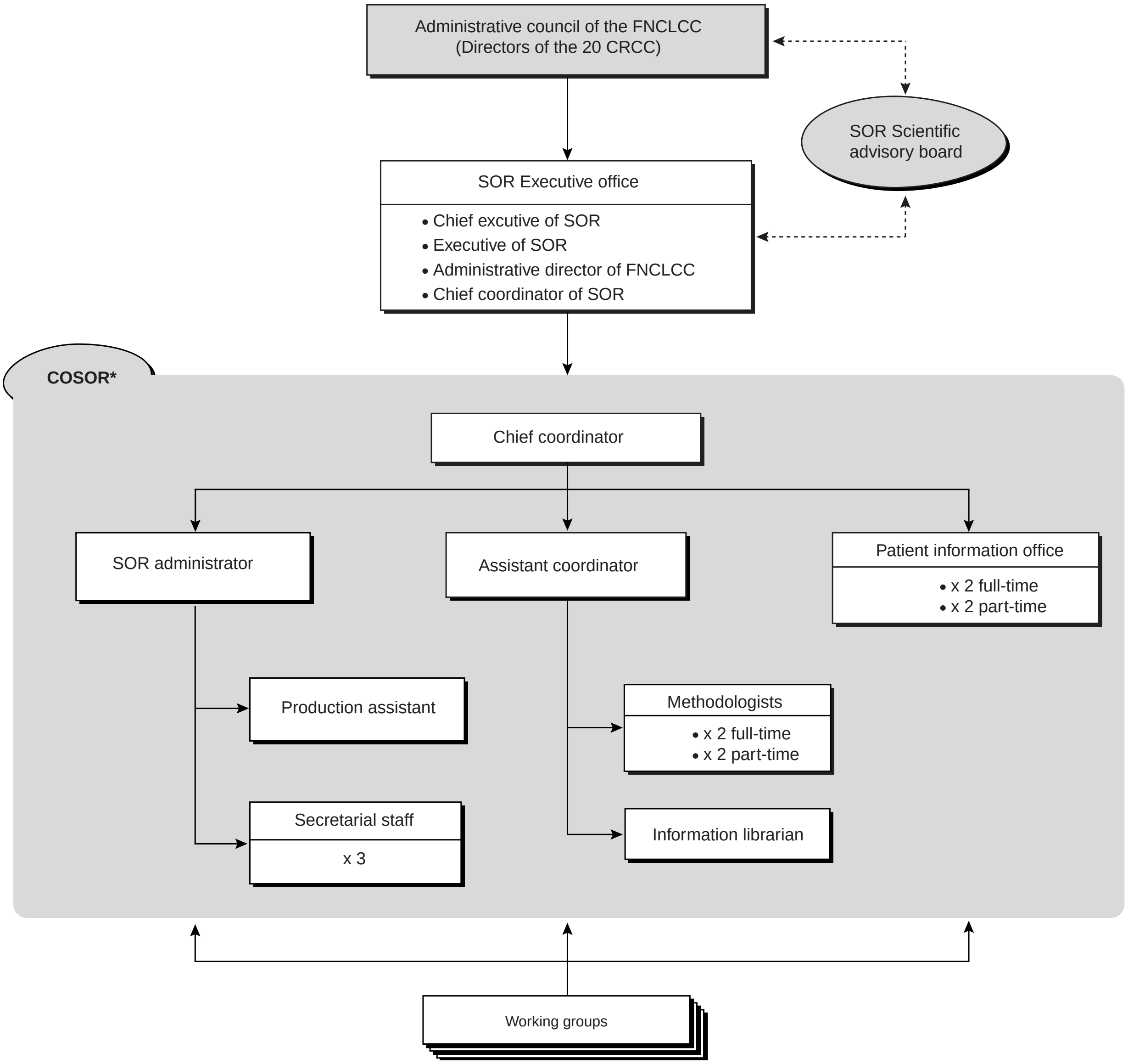

**Figure 1** Organization of the SOR project. COSOR = Organising Committee for SOR

### Literature search

The compilation of an exhaustive and relevant bibliography is a key element in the formation of the SORs. This is based primarily on published and indexed literature. The literature search is performed by a professional information librarian and is carried out according to standard procedures (Bonichon et al, 1998), including the search of electronic databases (Medline, Cancerlit), databases of systemic reviews (Cochrane library) and Internet sites of organizations developing and/or evaluating guidelines. Details of the strategy used for each SOR document are published in the full guideline report of each SOR.

### Critical appraisal of the evidence

Explicit criteria are used for the assessment of the validity of trial results and the quality of the methodology according to the type of publication (Guyatt et al, 1993, 1994, 1997; Oxman et al, 1993, 1994; Jaeschke et al, 1994a, 1994b; Laupacis et al, 1994; Levine et al, 1994; Hayward et al, 1995). To improve methodological rigor, to limit bias in the selection of relevant studies and to facilitate the data review process, critical appraisal checklists have been developed for different types of publications (Fervers, 2000). The aim has been to achieve a balance between practicability and methodological rigor while acknowledging the time constraints of experts. The critical appraisal checklists contain the criteria necessary for the assessment of the quality and the clinical relevance of the information (Figure 2). Experts are required to state the reason if any evidence is excluded.

### Synthesis of the evidence

The relevant studies are tabulated to present the study method and population, and the benefits and potential disadvantages of each

| **Reference:** |
|---|

| **Question** | **Yes** | **No** | **Not possible to determine or not applicable** |
|---|---|---|---|
| 1. Is the study randomized? | | | |
| 2. Is it a prospective study? | | | |
| 3. Is it a retrospective study? | | | |
| 4. Are the primary end-points defined? | | | |
| 5. Is the time of evaluation specified? | | | |
| 6. Is the number of patients included in the study specified? | | | |
| 7. Is the number of patients included in the analysis specified? (the results relate to only a sub-section of patients included) | | | |
| 8. Is the number of patients required specified? | | | |
| 9. Have the authors answered the question initially asked according to the specified end-points? | | | |
| 10. Have all the patients been analysed in the group to which they were initially allocated whether or not they completed treatment?('Intention-to-treat' analysis) | | | |

| |
|---|
| 11. Is the quality of the study generally satisfactory?<br>If not, explain why. |
| 12. Are the results of this study to be included in the report or tables of a SOR document?<br>☐ **YES**<br>In the case of an up-date, do the results change the standards, options or recommendations of the SOR? ☐ **NO** ☐ **YES** in what way?<br>☐ **NO**, Why? |

*© 2001 FNCLCC*

*Put NS if not specified in the publication*

| **Reference:** | |
|---|---|
| Patient characterstics (stage, age, etc) | |

| | **arm A** | **arm B** | **arm C** | **arm D** |
|---|---|---|---|---|
| Description of treatment in each study arm | | | | |
| Number of patients included | | | | |
| Number of patients evaluated | | | | |
| Primary end-points | | | | |
| • Tumour volume *(complete or partial response)*<br>☐ partial response (RP) in %<br>☐ complete response (CR) in % | | | | |
| • Survival (%, duration)<br>☐ relapse free<br>☐ without local recurrence<br>☐ without metastases<br>☐ event free<br>☐ overall<br>(specify details) | | | | |
| • other, specify | | | | |
| Survival definitions<br>*(median, mean or survival at x years)* | | | | |
| Confidence intervals of primary end-point | | | | |
| *P* value of primary end-point | | | | |
| Definition of toxicity | | | | |
| Comments | | | | |

**Figure 2** (**A**) Critical appraisal checklist for therapeutic studies (**B**) data extraction form

*© 2001 FNCLCC*

intervention. This is accompanied by comments and conclusions as to the validity of the results. This facilitates a comparison of the various interventions and provides the basis for the formulation of the SORs.

### Formulation of the 'Standards, Options and Recommendations'

Based on the synthesis of the evidence, experts define the 'Standards', 'Options' and 'Recommendations' for a given clinical situation. These are based on the best available evidence for that condition. The differentiation of 'Standards' and 'Options' identifies clinical situations where there exist strong indications or contra-indications for a particular intervention (Standards) and situations where there are several alternatives, none of which have shown clear superiority over the others (Options) (Table 1).

In any SOR, there can be several 'Options' for one clinical situation. 'Recommendations' allow for the weighting of Options according to the available evidence. Several interventions can be recommended for the same clinical situation. In this way, clinicians can make a choice according to specific clinical parameters, e.g. local circumstances, skills, equipment, resources and patient preferences. This ability to adapt the SOR according to local situations is acceptable if the reason for the choice is sufficiently transparent and is crucial for successful implementation. Inclusion of patients in clinical trials is an appropriate form of patient management in oncology and is recommended frequently within the SORs, in particular in situations where the evidence to support an intervention is weak.

In order to give the user information about the type of evidence underlying any 'Standard', 'Option' or 'Recommendation', a classification of different levels of evidence was developed by the FNCLCC based on previously published models (Canadian Task Force on the Periodic Health Examination, 1988; Agence Nationale pour le Développement de l'Evaluation Médicale, 1993; Sackett, 1989; American Society of Clinical Oncology, 1994; Agency for Health Care Policy and Research, 1992). The level of evidence depends not only on the type and quality of the studies reviewed, but also on the concordance of the results (Table 2). When there is no clear scientific evidence, a judgement is made according to the professional experience and consensus of the expert group (expert agreement). The latter must be confirmed by independent review (see below). Experience has shown that classifications of levels of evidence have limitations and risk providing positive support for interventions based on poor-quality evidence (Woolf et al, 1996; Fervers et al, 1998a). Therefore, the use of any level of evidence needs to be accompanied by an explicit and transparent process for the reporting of the underlying evidence.

### Structure of the SOR report

All documents follow the same format with the critical appraisal of the scientific data and summary tables for each clinical question grouped into sections corresponding to diagnosis, staging, treatment and follow-up. At the end of each section a summary presents the Standards, Options and Recommendations, with accompanying levels of evidence.

### Clinical algorithms

The SORs are integrated into clinical pathways that are presented in the form of algorithms or decision trees (Figure 3). The algorithms are used as an aid for decision making (Hadorn et al, 1992). They provide a graphic representation of a situation, focusing on the specific clinical decisions that must be made at

**Table 1** Definitions

| |
|---|
| Standards<br>Procedures or treatments for which results are known and which are considered of benefit, inappropriate or harmful by unanimous decision<br>These represent strong indications or contraindications |
| Options<br>Methods for which the results of studies are known and which are considered of benefit, inappropriate or harmful by a majority<br>They represent relative indications or contraindications |
| Recommendations<br>These are the decisions and choices made by the expert panel and the reviewers according to the different methods of evaluation<br>They are used to rank the interventions by level of evidence when several methods are considered equally beneficial with respect to the same problem |

**Table 2** Definition of level of evidence

| |
|---|
| Level A<br>There exists a meta-analysis of high standard or several randomized therapeutic trials of high standard which given consistent results |
| Level B<br>There exist studies, therapeutic trials, quasi-experimental trials, or comparisons of populations, of which the results are consistent when considered together |
| Level C<br>There exist studies, therapeutic trials, quasi-experimental trials or comparisons of populations, of which the results are not consistent when considered together |
| Level D<br>Either the scientific data does not exist or there is only a series of cases |
| Expert agreement<br>The data does not exist for the method concerned but the experts are unanimous in their judgement |

© 2001 FNCLCC

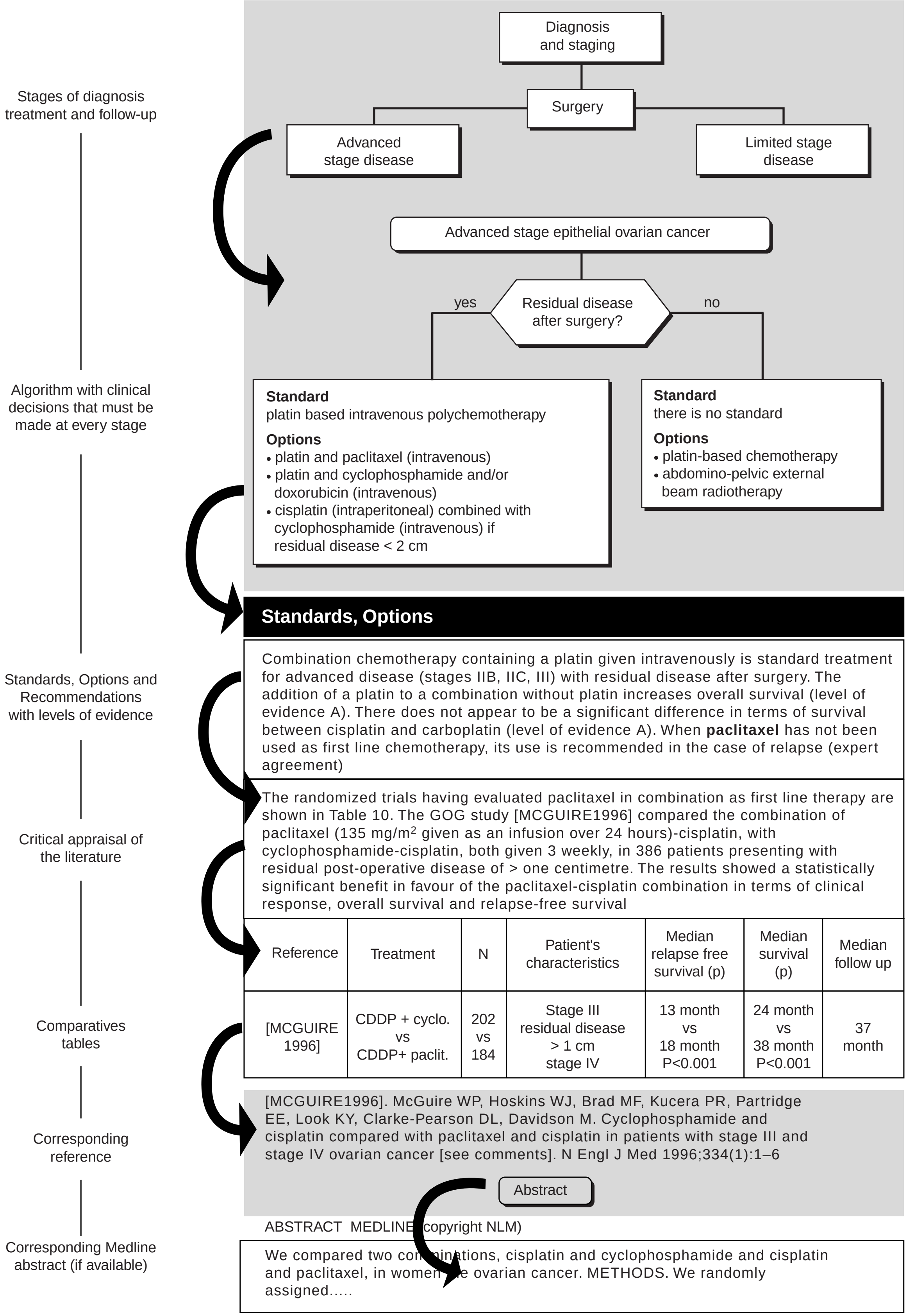

**Figure 3** Methods of access to different levels of information of SOR, example algorithm (epithelial ovarian cancer)

each stage in the management of a particular disease, that is in prevention, diagnosis, staging, treatment and follow-up (Margolis, 1983). The development of these 'decision trees' is an integral part of the guideline process and a key element of the SOR document (Grol et al, 1998). They facilitate the identification of the principle Standards and Options and refer directly to the relevant literature appraisal in the SOR document to give, as required, more details regarding the recommended practices (Figure 3). Along with the summaries of each section, the decision trees give a convenient practical guide for the management of each major tumour type.

### Independent review

This is undertaken using specific criteria developed for systematic reviews (Mulrow, 1987), as well as quality criteria referring more specifically to the guideline development and reporting process (Institute of Medicine, 1992; Cluzeau et al, 1999; AGREE Collaborative Group, 2000).

A questionnaire is sent to appropriate specialists who have not been involved in the guideline development process within the CRCCs, in relevant scientific societies and in the public and private sectors (Figure 4). They are asked to evaluate the quality of the SORs and whether they are in agreement with them. In the case of comments or reservations, a detailed written justification is requested, calling on relevant references from the literature and/or experience of the doctor. The relevance and the scientific data to support suggested changes are reviewed by the working group. In the case of major disagreement on an important topic, a second review is organized. The review process requires approval of the SOR documents by the medical committee in each CRCC.

### Guideline dissemination

The SOR documents are published in electronic form as a CD-ROM and in print in the form of monographs and articles. Some are available on the Internet (www.fnclcc.fr). The documents are sent to all CRCCs via each director, to practitioners who provided feedback, to members of collaborating scientific societies within and outside the CRCCs and to coordinators of regional cancer networks. Pharmaceutical companies are sent copies for distribution outside the CRCCs. Information on new SORs and the SOR catalogue are sent regularly by Internet mailing and to cancer centres, medical journals, the health department, etc. The recommendations are presented regularly at conferences and meetings.

To date, CD-ROM has been the main vehicle for dissemination. A navigation method has been developed, based on the structure of the SOR documents and the clinical algorithims. This facilitates a problem-based approach using the clinical algorithms as the main entry-point into the document, with hypertext links giving access to the different levels of information ranging from the most straightforward to the most complex. This is possible because of the identical structure of each of the SOR documents (Figure 3). When it exists, the corresponding Medline abstract can be accessed. The project is indebted to the National Library of Medicine and the National Institute of Health and of Medical Research (INSERM) who have authorized the use of Medline abstracts in the SOR CD-ROM. Publication details are listed in Appendix 1.

### Guideline update

Updating of clinical guidelines is a continuous process crucial for the integration of new evidence. The update process is based on literature searches performed on a regular basis. Depending on the type of evidence identified and its consistency with the evidence used to define the current SOR, different update strategies have been defined. Identification of data that may have a major impact on current guidelines will result in an 'urgent updating process'. 'Global updating' is performed on a regular basis and allows for the integration of new evidence as it becomes available. The updating process also focuses on specific clinical questions of high priority. A mail survey is undertaken to seek views of practitioners in order to prioritise topics to be addressed in the update process. A formal questionnaire has been developed to facilitate the process in which key points (e.g. severity and frequency of the target condition, uncertainty about appropriate practice, controversy regarding interpretation of data) are scored.

### Sources of funding

The SOR program has an annual budget of 6.9 million French francs (1.05 million Euros) and is funded primarily by the FNCLCC through contributions from each of the CRCCs and by grants from a major cancer charity, the National League against Cancer. Other sources of funding include the university hospitals, Ministry of Health, private clinics and scientific societies. Private industry has no financial, organizational or scientific participation in the guideline development process, although financial contributions are received from pharmaceutical companies to aid the dissemination of the SOR documents outside the CRCCs. The work of all experts is voluntary. There is no personal remuneration for the formulation or review of the SOR documents.

### ACHIEVEMENTS TO DATE

More than 1700 practitioners (doctors, pharmacists and biologists) have contributed to the project as members of working groups or as reviewers; 43% from the CRCCs, 43% from universities or general hospitals and 14% from the private sector (Fervers et al, 1998b). A total of 45 guidelines have been developed and published since 1993. The first CD-ROM was produced in 1995. A second CD-ROM containing new chapters plus updated versions published in 1998 had the equivalent of 5000 text pages including 740 tables, 270 clinical algorithms, 9560 bibliographic references, 5140 abstracts and 26 000 hypertext links. More than 5000 CD-ROMs were distributed in 1998. A total of 10 monographs, focusing on specific topics, have been published since (see Appendix 1).

Four thousand copies of the monographs of the most frequent cancers (colon, rectum, breast cancer and ovarian cancer) have been distributed. SORs are published every second month in the *Bulletin of Cancer* and in other French medical journals in collaboration with the French Society of Cancer. A number of SORs are being translated into English for submission to international journals.

Full versions of all documents are available from the FNCLLC. All SORs will soon be accessable on the FNCLCC web site (www.fnclcc.fr).

The program has been judged as successful on the basis of user awareness and utilization. A formal survey designed to evaluate

*© 2001 FNCLCC*

| Chapter:------------------------------------------------ | I am ...... In complete agreement | In general agreement | Without opinion? | In some disagree-ment | In complete disagree-ment | Comments: (please expand on your comments and attach to form) |
|---|---|---|---|---|---|---|
| 1) The methodology used for the development of the SOR is clearly presented | ☐ | ☐ | ☐ | ☐ | ☐ | -------------------------- |
| 2) The working group includes representatives from all relevant specialities for the present subject | ☐ | ☐ | ☐ | ☐ | ☐ | -------------------------- |
| 3) The sources of information are relevant and valid | ☐ | ☐ | ☐ | ☐ | ☐ | -------------------------- |
| 4) The subject is approached in a relevant fashion (plan, contents) | ☐ | ☐ | ☐ | ☐ | ☐ | -------------------------- |
| 5) All controversial issues have been addressed | ☐ | ☐ | ☐ | ☐ | ☐ | -------------------------- |
| 6) The population of the patients for whom the SOR is intended has been clearly specified | ☐ | ☐ | ☐ | ☐ | ☐ | -------------------------- |
| 7) The synthesis of the published data is clear | ☐ | ☐ | ☐ | ☐ | ☐ | -------------------------- |
| 8) There is an explicit link between the recommendations and the supporting level of evidence | ☐ | ☐ | ☐ | ☐ | ☐ | -------------------------- |
| 9) Any exceptions are clearly stated | ☐ | ☐ | ☐ | ☐ | ☐ | -------------------------- |
| 10) The various options for the management, drug therapy and/or best practice in the particular disease are clearly presented | ☐ | ☐ | ☐ | ☐ | ☐ | -------------------------- |
| 11) The presentation/format of the SOR is clear and unambiguous | ☐ | ☐ | ☐ | ☐ | ☐ | -------------------------- |
| 12) The potential benefits, risks and side effects of the interventions have all been considered in formulating the recommendations | ☐ | ☐ | ☐ | ☐ | ☐ | -------------------------- |
| 13) It is confimed that there are no other possible conclusions that could be made from the available data | ☐ | ☐ | ☐ | ☐ | ☐ | -------------------------- |
| 14) The clinical situations in which the SOR can be applied has been made clear | ☐ | ☐ | ☐ | ☐ | ☐ | -------------------------- |
| 15) All the relevant available literature has been considered | ☐ | ☐ | ☐ | ☐ | ☐ | -------------------------- |

| | Entirely | Mostly (detail) | To some extent (detail) | Not at all | |
|---|---|---|---|---|---|
| 16) This SOR seems applicable to my clinical practice | ☐ | ☐ | ☐ | ☐ | -------------------------- |
| 17) I approve of the SOR for the stated subject | ☐ | ☐ | ☐ | ☐ | -------------------------- |
| 18) I agree to use these recommendations in my clinical practice | ☐ | ☐ | ☐ | ☐ | -------------------------- |

**Figure 4** Checklist for independent review of SOR documents dissemination, awareness and the use of the guidelines involving representative cancer specialists from the 20 CRCCs was undertaken in 1998. This showed a high rate of awareness of the SOR program (98.3%) and that the SOR documents had been disseminated widely. Ninety percent of specialists surveyed had received the SOR guidelines. The majority (95%) of the practitioners used the SOR in practice, during multidisciplinary (33%) or individual (27%) decision-making processes. The SORs were also being used as a source of information for teaching (20%) and for finding evidence (20%).

### CONCLUSION

The aim of the SOR project is to provide cancer care specialists with information on the current best clinical practice in a given situation, based on a synthesis of the available evidence by a multidisciplinary expert panel.

The project has evolved over time as a consequence of practitioner-led initiatives to achieve a balance between methodological rigor and practicability. CPGs cannot replace clinical reasoning based on experience. An explicit and transparent approach in the guideline development and reporting process is necessary to show how underlying ‘evidence judgements’ and ‘preference judgements’ have been made (Eddy, 1992). This is particularly important for topics in which the evidence is weak.

Routine implementation of such guidelines remains a major challenge. Implementation of SORs at local and regional level has been shown to be effective (Ray-Coquard et al, 1997). However, there is currently no mechanism for the routine implementation of SORs. Representatives of all potential users at national level are encouraged to become involved in the project as early as possible to improve acceptance and implementation. Implementation strategies have to take into account local circumstances (Davis et al, 1995; Oxman et al, 1995). The adoption of the SOR guidelines by clinicians at local and regional level is an important step in successful implementation. This is one of the major aims of the regional cancer network of CRCCs in France.

### REFERENCES

Agence Nationale pour le Développement de l’Evaluation Médicale (1993) *Les recommendations pour la pratique clinique. Guide pour leur élaboration*. ANDEM: Paris

Agency for Health Care Policy and Research (1992). *Agency for Health Care Policy and Research Guideline Report*. AHCPR: Rockville

AGREE Collaborative Group (2000) Guideline development in Europe: an international comparison. *Int J Technol Assess Health Care* **16**(4): 1036–1046.

American Society of Clinical Oncology (1994) Recommendations for the use of hematopoietic colony-stimulating factors: evidence-based, clinical practice guidelines. *J Clin Oncol* **12**: 2471–2508

Bonichon F, Courtial F and Blanc-Vincent MP (1998) Revue de la littérature, recherche d’informations et lecture critique d’articles en cancérologie. *Bull Cancer* **35**: 86–885

© 2001 FNCLCC

Browman GP, Levine MN, Mohide EA, Hayward RS, Pritchard KI, Gafni A and Laupacis A (1995) The practice guidelines development cycle: a conceptual tool for practice guidelines development and implementation. *J Clin Oncol* **13**: 502–512
Canadian Task Force on the Periodic Health Examination (1988) The periodic health examination: 2. 1987 update. *CMAJ* **138**: 618–626
Cluzeau FA, Littlejohns P, Grimshaw JM, Feder G and Moran SE (1999) Development and application of a generic methodology to assess the quality of clinical guidelines. *Int J Qual Health Care* **11**: 21–28
Davis DA, Thomson MA, Oxman AD and Haynes RB(1995) changing physician performance. A systematic review of the effect of continuing medical education strategies [see comments]. JAMA 274: 700–705
Eddy DM (1992) A Manual for Assessing Health Practices and Designing Practice Policies. American College of Physicians: Philadelphia
Fervers B (2000) Les recommandations pour la pratique clinique: l'exemple des Standards, Options et Recommandations de la FNCLCC. In *Nouvelle évaluation médicale*, Giraud A, Boissel JP, Fervers B, Grenier B, Launois R and Leplège A (eds) Economica: Paris
Fervers B, Bonichon F, Demard F, Héron JF, Mathoulin S, Philip T, Renaud-Salis JL, Toma C and Bey P (1995) Méthodologie de développement des standards, options et recommandations diagnostiques et thérapeutiques en cancérologie. *Bull Cancer* **82**: 761–767
Fervers B, Carrère MO, Späth HM and Philip T (1998a) Intégration des critéres économiques dans les recommandations pour la pratique clinique. *Bull Cancer* **85**: 272–280
Fervers B, Bey P, Philip T, Blanc-Vincent MP, Farsi F Labrèze L, Lehmann M, Sarradet A and Théobald S (1998b) Standards, options et recommandations: un espace dans le Bulletin du Cancer pour faciliter leur diffusion. *Bull Cancer* **85**: 149–151.
Grol R, Dalhuijsen J, Thomas S, Veld C, Rutten G and Mokkink H (1998) Attributes of clinical guidelines that influence use of guidelines in general practice: observational study. *BMJ* **317**: 858–861
Guyatt GH, Sackett DL and Cook DJ (1994) Users' guides to the medical literature. II. How to use an article about therapy or prevention. B. What were the results and will they help me in caring for my patients? Evidence-Based Medicine Working Group. *JAMA* **271**: 59–63
Guyatt GH, Naylor CD, Juniper E, Heyland DK, Jaeschke R and Cook DJ (1997) Users' guides to the medical literature. XII. How to use articles about health-related quality of life. Evidence-Based Medicine Working Group. *JAMA* **277**: 1232–1237.
Hadorn DC, McCormick K and Diokno A (1992) An annotated algorithm approach to clinical guideline development. *JAMA* **267**: 3311–3314
Hayward RS, Wilson MC, Tunis SR, Bass EB and Guyatt G (1995) Users' guides to the medical literature. VIII. How to use clinical practice guidelines. A. Are the recommendations valid? The Evidence-Based Medicine Working Group. *JAMA* **274**: 570–574
Institute of Medicine, Committee on Clinical Practice Guidelines and Division of Health Care Services (1992) *Guidelines for clinical practice: From development to use*. National Academic Press: Washington DC
Jaeschke R, Guyatt GH and Sackett DL (1994a) Users' guides to the medical literature. III. How to use an article about a diagnostic test. B. What are the results and will they help me in caring for my patients? The Evidence-Based Medicine Working Group. *JAMA* **271**: 703–707
Jaeschke R, Guyatt G and Sackett DL (1994b) Users' guides to the medical literature. III. How to use an article about a diagnostic test. A. Are the results of the study valid? Evidence-Based Medicine Working Group. *JAMA* **271**: 389–391
Laupacis A, Wells G, Richardson WS and Tugwell P (1994) Users' guides to the medical literature. V. How to use an article about prognosis. Evidence-Based Medicine Working Group. *JAMA* **272**: 234–237
Levine M, Walter S, Lee H, Haines T, Holbrook A and Moyer V (1994) Users' guides to the medical literature. IV. How to use an article about harm. Evidence-Based Medicine Working Group. *JAMA* **271**: 1615–1619
Margolis CZ (1983) Uses of clinical algorithms. *JAMA* **249**: 627–632
Mulrow CD (1987) The medical review article: state of the science. *Ann Intern Med* **106**: 485–488
Négrier S, Bailly C, Beckendorf V, Cupissol D, Doré JF, Dorval T, Fervers B, Garbay JR and Vilmer C Fédération Nationale des Centres de Lutte Contre le Cancer (1998) *Mélanome cutané*. John Libbey EUROTEXT: Paris
Oxman AD, Sackett DL and Guyatt GH (1993) Users' guides to the medical literature. I. How to get started. The Evidence-Based Medicine Working Group. *JAMA* **270**: 2093–2095
Oxman AD, Cook DJ and Guyatt GH (1994) Users' guides to the medical literature. VI. How to use an overview. Evidence-Based Medicine Working Group [see comments]. *JAMA* **272**: 1367–1371
Oxman AD, Thomson MA, Davis DA and Haynes RB (1995) No magic bullets: a systematic review of 102 trials of interventions to improve professional practice. *CMAJ* **153**: 1423–1431
Ray-Coquard I, Philip T, Lehmann M, Fervers B, Farsi F and Chauvin F (1997) Impact of a clinical guidelines program for breast and colon cancer in a French cancer center. *JAMA* **278**: 1591–1595
Sackett D (1989) Rules of evidence and clinical recommandations on the use of antithrombotic agents. Chest 95 (suppl 2): 2S–4S.
Woolf SH (1992) Practice guidelines, a new reality in medicine. II. Methods of developing guidelines. *Arch Intern Med* **152**: 946–952
Woolf SH, DiGuiseppi CG, Atkins D and Kamerow DB (1996) Developing evidence-based clinical practice guidelines: Lessons learned by the US preventive services task force. *Annu Rev Public Health* **17**: 511–538

 *© 2001 FNCLCC*
